# Retrospective analysis of crescent score in clinical prognosis of IgA nephropathy

**DOI:** 10.1515/med-2022-0414

**Published:** 2022-01-24

**Authors:** Ying Chen, Yiya Yang, Yumei Liang, Manting Liu, Wei Xiao, Xiaofang Hu

**Affiliations:** Department of Nephrology and Laboratory of Kidney Disease, Hunan Provincial People’s Hospital, The First Affiliated Hospital of Hunan Normal University, Changsha Clinical Research Center for Kidney Disease, Hunan Clinical Research Center for Chronic Kidney Disease, Changsha, Hunan 410005, China; Department of Urology, Hunan Provincial People’s Hospital, The First Affiliated Hospital of Hunan Normal University, Changsha, Human 410005, China; Department of Internal Medicine, Hunan Normal University School of Medicine, Changsha, Hunan 410013, China

**Keywords:** IgA nephropathy, Oxford classification, crescent score, renal prognosis

## Abstract

The scoring of crescents (Cs) was recently added to the Oxford classification for IgA nephropathy (IgAN). Because of the short-term use of the C score in clinical practice, its validity and applicability need to be verified. We, retrospectively, analyzed the clinicopathological data of 144 primary IgAN patients diagnosed at our hospital from March 2017 to March 2019 and with complete ≥6-month follow-up data. We found that the C score was positively correlated with the Lee’s classification in the assessment of renal pathological changes and significantly correlated with increased proteinuria and decreased estimated glomerular filtration rate. Univariate Cox regression analysis showed an association of C formation with IgAN prognosis, and multivariate Cox regression indicated Cs as an independent prognosis factor. The optimal proportion of Cs for prognosis prediction by the receiver operating characteristic curve was 11%. Kaplan–Meier survival curve revealed a significantly decreased renal survival rate in patients with C proportions ≥11%. Further multivariate Cox regression analysis confirmed that the C proportion ≥11% is an independent risk factor for poor prognosis of IgAN patients. Our findings demonstrate that Cs are independently related to the prognosis of patients with IgAN, and the proportion of Cs ≥11% is an independent risk factor for poor outcomes.

## 1 Introduction

IgA nephropathy (IgAN) is a common glomerulonephritis that can cause end-stage renal disease (ESRD). IgAN is diagnosed clinically by histopathological examination of renal biopsy tissues with the mesangial deposits of IgA and related immune complexes [1]. IgAN can occur at any age and has highly variable clinical manifestations, such as hematuria with varying degrees of proteinuria, hypertension, and impaired renal function. Certain systemic disorders, such as Henoch–Schonlein purpura nephritis and systemic lupus erythematosus, can also lead to IgA deposition in the glomerular mesangium, which is called secondary IgAN [2].

Due to the clinical and pathological diversity of IgAN, this disease progression and prognosis varies interindividually. Therefore, it is necessary to ascertain the risk factors that influence the progression and prognosis of IgAN. In 1982, Lee et al. sorted IgAN patients into the grades 1 through 5 of pathological damage (i.e., mesangial cell proliferation, glomerulosclerosis, C formation, and tubulointerstitial alteration) [3]; the higher the grade of IgAN, the more severe the disease is and the shorter the survival is. Because Lee’s grading for IgAN is simple and easy to operate, it is useful in guiding treatment choices and predicting clinical outcomes [4,5,6]. However, this classification has some shortcomings, mainly lacking an objective evaluation of pathological manifestations [7], which may lead to a biased prediction of prognosis. This has, however, been improved with the introduction of the Oxford classification in 2009, which proposed four highly reproducible variables that can independently predict prognosis, namely mesangial hypercellularity (M), endocapillary proliferation (E), segmental glomerulosclerosis (S), and tubular atrophy/interstitial fibrosis (T) [8,9].

Crescents (Cs) are a common pathological lesion in IgAN, occurring in approximately 18.8–66.4% of renal biopsy specimens. Glomerular Cs begin with cellular C, which gradually transforms into cellular/fibrous C and then into irreversible fibrous C, and finally manifests as glomerulosclerosis, causing permanent renal damage and even ESRD [10,11]. C formation is closely related to various clinicopathological features; it has a positive correlation with proteinuria and serum creatinine (Scr) [12] and is also associated with global sclerosis, segmental glomerulosclerosis, endocapillary proliferation, and renal tubulointerstitial lesions. Therefore, though controversial, C formation is regarded as an important prognostic marker for IgAN. In 2016, Haas et al. [13] in a study of 3,096 patients with IgAN confirmed that C formation is an independent risk factor for poor prognosis in patients with IgAN. The risk of renal progression for patients with Cs in ≥25% of glomeruli is much higher than that of patients with a proportion of Cs <25%. Due to the independent prognostic significance of Cs, the revised Oxford classification (2017) has included Cs by dividing into C0 (no Cs), C1 (<25% of glomeruli containing Cs), and C2 (≥25% of glomeruli with Cs) [14].

Nevertheless, because of the short-term use of the C score in clinical practice, its validity and applicability remain to be verified. In this study, we collected the clinicopathological data of patients with primary IgAN and compared the C scoring to the Lee’s grading system in assessing the pathological changes. We also evaluated the significance of the C score in predicting renal outcome in IgAN, particularly determining the optimal cutoff value of C proportion that best correlates with patient prognosis.

## 2 Methods

### 2.1 Subjects

This was a retrospective investigation of patients with primary IgAN diagnosed at Hunan Provincial People’s Hospital from March 2017 to March 2019. The diagnosis was based on renal biopsy findings of IgA or IgA-dominant deposits in the glomerular mesangium and possibly in the capillary loops under immunofluorescence microscopy. Inclusive criteria: (1) aged ≥14 years, with complete ≥6 month clinical follow-up information; (2) at biopsy, total number of glomeruli per section ≥8; (3) having no other kidney complications, for example, diabetic nephropathy; (4) without systemic diseases, for example, systemic lupus erythematosus, infection, active tuberculosis, tumor, and cachexia; and (5) without the presence of secondary IgAN (e.g., hepatitis B-related nephritis, liver cirrhosis, and Henoch–Schonlein purpura nephritis).

In this context, 158 patients were diagnosed with primary IgAN after excluding those with renal complications, systemic diseases, and secondary IgAN in 512 patients who underwent renal biopsy. As 14 primary IgAN cases had incomplete clinical follow-up data, they were also excluded. Finally, a total of 144 patients were included in this study. As of September 30, 2019, the longest follow-up interval was 30 months.

### 2.2 Clinical data collection

Clinical data were obtained by retrieving medical records and follow-up data including sex, age (at the time of kidney biopsy), blood pressure, mean arterial pressure (MAP), 24 h urine protein, estimated glomerular filtration rate (eGFR), Scr, urea nitrogen (BUN), uric acid (UA), serum albumin (ALB), hemoglobin (HGB), total cholesterol (TC), triglycerides (TG), high density lipoprotein (HDL), low density lipoprotein (LDL), serum IgA, serum C3, and serum C4.

By using the Modification of Diet in Renal Disease Study equation (2005 version) [15], eGFR (mL/min/1.73 m^2^) = 186 × [Scr (μmol/L)/88.4]^−1.154^ × age^−0.203^ × 0.742 (female). MAP (mmHg) = diastolic blood pressure (mmHg) + (systolic blood pressure (mmHg) − diastolic blood pressure (mmHg))/3. The 24 h urinary protein quantification (g/24 h) = urine protein (g/L) × 24 h urine volume (L/24 h).

### 2.3 Pathological data collection

All renal biopsies were performed in our hospital between March 2017 and March 2019. These biopsy specimens were cut into 2–3 μm sections and stained with conventional hematoxylin and eosin, periodic acid-Schiff, periodic acid-silver methenamine, and Masson’s Trichrome. They were then forwarded to two pathologists who did not know the patients’ data for diagnosis. We obtained pathological data by reviewing the biopsy records, and reassessed the pathological gradings according to the 2017 Oxford classification system, notably the C lesions (cellular C, fibro-cellular C, and fibrous C) and C proportion. By reexamining the biopsy sections and reaching a consensus, the difference in results between the two pathologists was resolved.

### 2.4 Definitions

The primary renal endpoint was defined as the patient entering ESRD or eGFR decreasing by 50%. The duration from the diagnosis by kidney biopsy to the occurrence of the endpoint event was defined as the kidney survival time.

### 2.5 Statistical analysis

SPSS 24.0 software (IBM, Chicago, IL, USA) was used for statistical analysis. Spearman rank correlation coefficient was used to analyze the correlation between the C score and the Lee’s grading. Clinical quantitative parameters with normal distribution are expressed as the mean ± standard deviation and compared by using the Student *t*-test while variables with nonnormal distribution are expressed as median (interquartile range) and analyzed by using the Wilcoxon signed rank test. For categorical variables, the data are expressed as numbers (percentages), and the Chi-square test was used. Univariate and multivariate Cox regression models were used to analyze the relationship between the classification and the renal endpoint event. The receiver operating characteristic (ROC) curve was used to determine the optimal cutoff value of C proportion that best correlates with patient prognosis. Kaplan–Meier survival curve was used in renal survival analysis. A *P* < 0.05 was considered statistically significant.


**Ethical approval and informed consent:** This study was carried out with the approval of the Ethics Committee of the Hunan Provincial People’s Hospital. An informed consent from patients with IgAN was not required by the ethics committee for this retrospective study.

## 3 Results

### 3.1 Correlation between the C score and Lee’s grading

Pathological classifications of 144 patients based on the Oxford MEST-C score and Lee’s grading are shown in Table 1. The distribution of these patients according to the crescentic lesions was C0, 59 (40.97%); C1, 77 (53.47%), and C2, 8 (5.56%). The distribution of patients according to the Lee’s grading was 4 (2.78%), 36 (25.00%), 67 (46.53%), 22 (15.28%), and 15 (10.42%) for grades I–V, respectively. Spearman correlation coefficient analysis shows that in addition to the MEST score, the C score was also positively correlated with the Lee’s grading (*r* = 0.654, *P* < 0.001; Table 2).

**Table 1:** Pathological classification of 144 patients with IgAN

Lee’s grading	No.	MEST-C score											
		M0	M1	E0	E1	S0	S1	T0	T1	T2	C0	C1	C2
I	4	4	0	4	0	4	0	4	0	0	4	0	0
II	36	11	25	36	0	34	2	36	0	0	36	0	0
III	67	9	58	41	26	52	15	64	3	0	13	51	3
IV	22	5	17	12	10	12	10	8	14	0	4	15	3
V	15	0	15	9	6	6	9	0	3	12	2	11	2
Total	144	29	115	102	42	108	36	112	20	12	59	77	8

**Table 2:** Correlation of the MEST-C score with Lee’s grading in patients with IgAN

Pathological classification		M	E	S	T	C
Lee’s grading	*r*	0.261	0.347	0.396	0.703	0.654
	*P*	0.002	<0.001	<0.001	<0.001	<0.001

*r*, Spearman correlation coefficient.

### 3.2 Relationship between the C score and clinical parameters

In agreement with earlier reports [8], we confirmed an association of the MEST score and clinical data of patients with IgAN (Tables A1–A4). Based on these data, we further evaluated the correlation between the C score and different clinical parameters (Table 3). As C2 had only eight patients, they were combined into C1. We found that C1/2 had a higher male composition (46.25%) than C0 (29.69%) (*P* < 0.05). Moreover, patients in the C1/2 had significantly higher levels of MAP, Scr, and 24 h urine protein, but lower levels of eGFR than those in the C0 group (all *P* < 0.05).

**Table 3:** Relationship of the C score with clinical parameters of patients with IgAN

Clinical parameter	C0 (*n* = 59)	C1/2 (*n* = 85)	*P*
Sex (male, %)	19 (29.69)	37 (46.25)	0.043*
Age (year)	34.27 ± 12.00	35.99 ± 11.99	0.363
Hypertension (*n*, %)	13 (20.31)	24 (30.00)	0.186
MAP (mmHg)	93.03 ± 12.80	99.76 ± 13.31	0.003*
Scr (μmol/L)	80.73 ± 42.84	107.58 ± 77.41	0.000*
eGFR (mL/min/1.73 m^2^)	99.77 ± 33.74	84.59 ± 31.05	0.028*
ALB (g/L)	39.62 ± 6.08	38.03 ± 5.68	0.113
24 h urine protein (g/24 h)	0.64 ± 0.93	1.61 ± 2.14	0.001*
BUN (mmol/)	5.02 ± 1.92	5.70 ± 2.99	0.667
UA (μmol/L)	338.7 ± 96.0	365.0 ± 106.6	0.248
TC (µmol/L)	4.37 ± 1.88	4.63 ± 1.17	0.305
TG (µmol/L)	1.60 ± 1.10	1.90 ± 1.40	0.169
HDL (µmol/L)	1.28 ± 0.32	1.25 ± 0.37	0.529
LDL (µmol/L)	2.67 ± 1.41	2.72 ± 0.91	0.823
HGB (g/L)	127.3 ± 14.9	125.5 ± 18.7	0.548
C3 (g/L)	0.99 ± 0.20	1.03 ± 0.21	0.240
C4 (g/L)	0.23 ± 0.71	0.27 ± 0.09	0.054
IgA (g/L)	3.20 ± 1.09	3.29 ± 1.00	0.677
Median follow-up period and range (months)	21 (6–30)	16 (6–30)	0.167

MAP, mean arterial pressure; Scr, serum creatinine; eGFR, estimated glomerular filtration rate; ALB, serum albumin; BUN, urea nitrogen; UA, uric acid; TC, total cholesterol; TG, triglycerides; HDL, high density lipoprotein; LDL, low density lipoprotein; and HGB, hemoglobin. All quantitative data are expressed as the mean ± standard deviation and categorical variables are expressed as number (percentage). *, statistically significant.

### 3.3 Relationship between the C score and patient prognosis

Of 144 patients, 17 (11.80%) reached the primary endpoint. The clinicopathological parameters that affect the patient prognosis are shown in Table 4. Univariate Cox regression analysis shows that sex, hypertension, MAP, Scr, BUN, UA, eGFR, 24 h urine protein, and TG as well as S, T, and C classifications were related to the prognosis of patients with IgAN (Table 4). Further analysis of potential independent prognostic factors (MAP, eGFR, and 24 h urine protein as well as E, S, T, and C) by using a multivariate Cox proportional hazards regression model confirmed that MAP, eGFR, S, T, and C are all independent factors affecting prognosis (all *P* < 0.05; Table 4).

**Table 4:** Cox regression analysis of prognostic factors for patients with IgAN

Parameter	Univariate		Multivariate	
	HR (95% CI)	*P*	HR (95% CI)	*P*
Sex	3.458 (1.263–9.472)	0.016*		
Age	1.020 (0.980–1.062)	0.333		
Hypertension	9.604 (3.353–27.509)	<0.001*		
MAP	1.074 (1.041–1.108)	<0.001*	1.051 (1.001–1.103)	0.046*
Scr	1.020 (1.014–1.026)	<0.001*		
BUN	1.377 (1.242–1.526)	<0.001*		
UA	1.007 (1.003–1.010)	<0.001*		
eGFR	0.920 (0.892–0.949)	<0.001*	0.933 (0.890–0.978)	0.004*
24 h urine protein	1.492 (1.271–1.748)	<0.001*	1.001 (0.694–1.445)	0.995
ALB	0.959 (0.902–1.020)	0.181		
HGB	0.977 (0.945–1.010)	0.167		
TC	1.159 (0.944–1.425)	0.159		
TG	1.320 (1.063–1.639)	0.012*		
M	4.947 (0.655–37.370)	0.121		
E	2.624 (0.960–7.168)	0.06	2.653 (0.441–15.953)	0.286
S	6.541 (2.393–17.882)	<0.001*	3.629 (1.063–12.385)	0.040*
T^a^	49.92 (11.04–225.60)	<0.001*	7.717 (1.148–51.861)	0.036*
C^b^	5.809 (1.637–20.616)	0.006*	5.090 (1.215–21.333)	0.026*

T^a^, T1 plus T2 vs T0; C^b^, C1/2 vs C0. *, statistically significant.

### 3.4 Optimal predictive value of Cs

**Figure 1:**
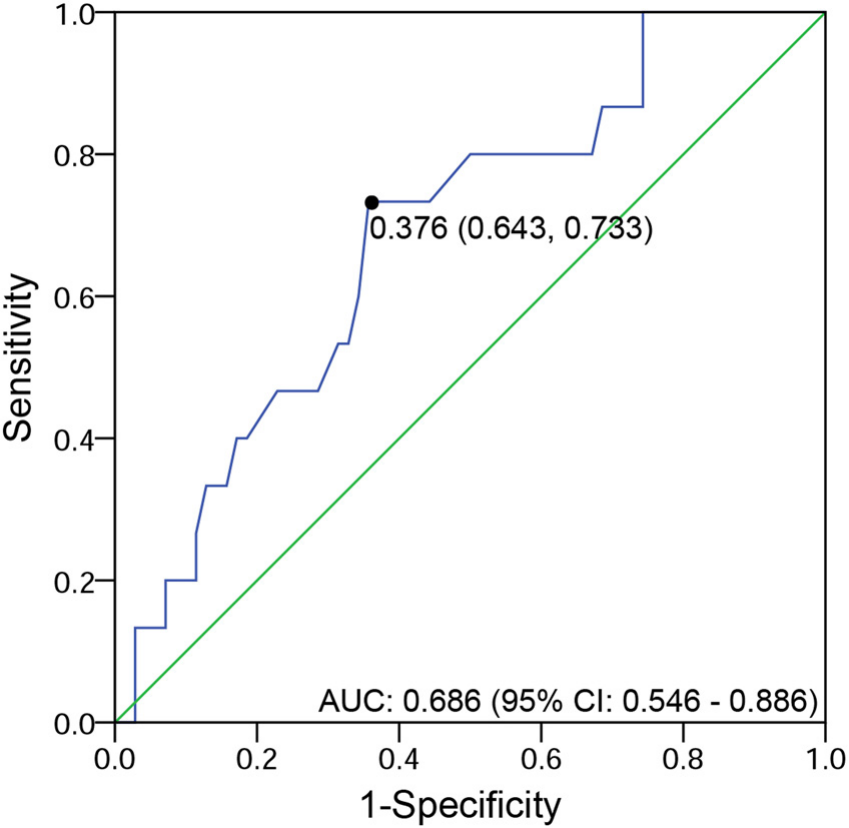
ROC curve analysis of the optimal C proportion for predicting renal survival.

Among all 144 patients, including 40.97% (59) C0 and 59.03% (85) C1/2, ROC curve analysis of 85 patients with crescentic lesions revealed that the optimal C proportion for predicting renal survival was 11% (The area under the curve = 0.686, sensitivity = 73.3%, and specificity = 64.3%; Figure 1).

### 3.5 Kaplan–Meier survival analysis of different C proportions

**Figure 2:**
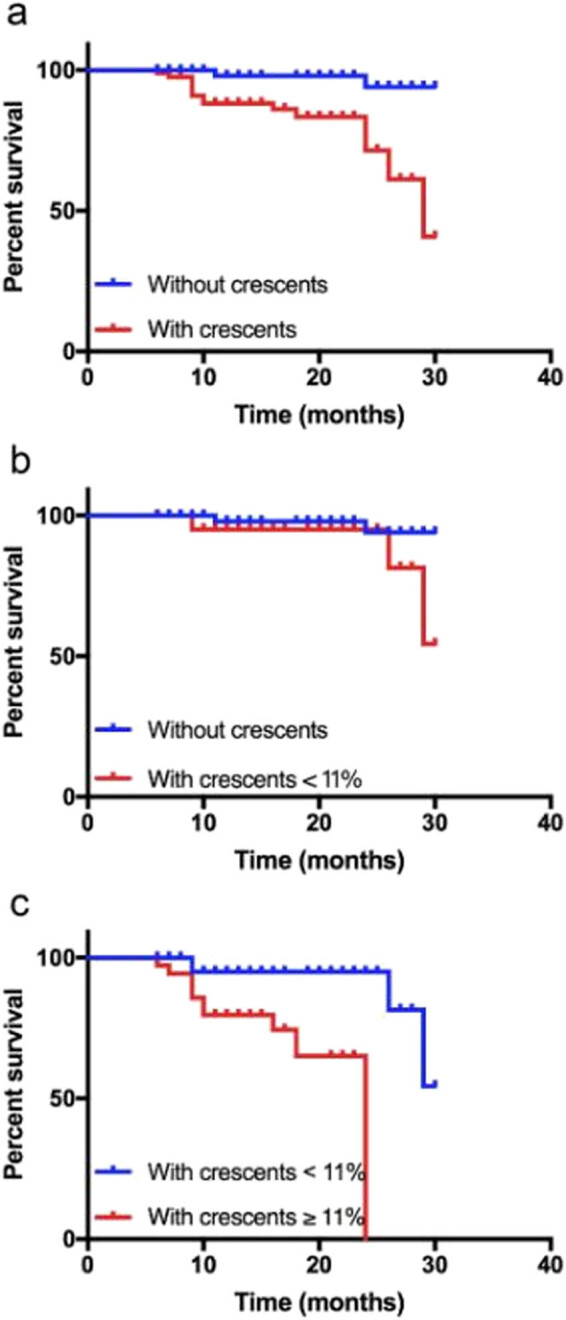
Kaplan–Meier survival analysis of IgAN patients with different C proportions: (a) C-free patients vs those with Cs, (b) C-free patients vs those with <11% of glomeruli containing Cs, and (c) patients with <11% of glomeruli containing Cs vs those ≥11% of glomeruli with Cs.

Based on the C proportion, 144 patients were divided into three groups: C-free, 59 (40.97%); <11%, 49 (34.03%); and ≥11%, 36 (25.00%). Accordingly, the distribution of 17 patients (11.80%) reaching the endpoint was C-free, 2 (1.39%); <11%, 4 (2.78%); and ≥11%, 11 (7.64%). Kaplan–Meier survival analysis shows that compared to the C-free group, patients with Cs (<11% plus ≥11%) had worse overall survival (*P* = 0.0009; Figure 2a). Moreover, there was no difference in survival between the C-free and <11% groups (Figure 2b); however, the ≥11% group had a worse survival than the <11% groups (*P* < 0.001; Figure 2c), demonstrating that C score is a good prognostic indicator, and patients with greater than the 11% cutoff value have a worse prognosis.

### 3.6 The C proportion ≥11% is an independent risk factor for IgAN prognosis

Finally, we analyzed the relationship between C proportion and IgAN prognosis by using the Cox proportional hazards regression model. Univariate Cox regression analysis shows that MAP, eGFR, and 24 h urine protein as well as S, T, and the ≥11% C proportion were highly associated with the prognosis of IgAN patients (all *P* < 0.05; Table 5). By selecting the ≥11% C proportion, MAP, eGFR, 24 h urine protein, E, S, and T for multivariate Cox proportional hazards regression analysis, we verified that the C proportion ≥11% is still an independent risk factor for poor prognosis of IgAN patients, and eGFR, S, and T are also associated with the prognosis of IgAN (all *P* < 0.05; Table 5).

**Table 5:** Cox regression analysis of the C score for prognostic prediction of IgAN patients

Parameter	Univariate		Multivariate	
	HR (95% CI)	*P*	HR (95% CI)	*P*
C-free	1.0			
Cs <11%	3.628 (0.660–19.943)	0.138		
Cs ≥11%	29.310 (5.356–160.385)	<0.001*	7.801 (1.399–43.482)	0.019*
MAP	1.074 (1.041–1.108)	<0.001*	1.053 (1.000–1.109)	0.052
eGFR	0.920 (0.892–0.949)	<0.001*	0.938 (0.894–0.984)	0.009*
24 h urine protein	1.492 (1.271–1.748)	<0.001*	1.022 (0.709–1.472)	0.908
M	4.947 (0.655–37.370)	0.121		
E	2.624 (0.960–7.168)	0.06	2.613 (0.407–16.781)	0.311
S	6.541 (2.393–17.882)	<0.001*	3.546 (1.041–12.080)	0.043*
T^a^	49.92 (11.04–225.60)	<0.001*	8.005 (1.148–55.833)	0.036*

Note(s): T^a^, T1/2 vs T0. *, statistically significant.

## 4 Discussion

To assess the clinical applicability of the C score, we, retrospectively, analyzed 144 primary IgAN patients. In this study, 59.03% of patients had C formation, which was in accordance with earlier findings by the original Oxford cohort [8]. Considering the effectiveness of the Lee’s grading in practice over the years, we first determined the correlation between the C score and the Lee’s grading. Spearman correlation analysis confirmed that C score is positively correlated with the Lee’s grading. However, we found that each pathological index in the Oxford classification has inconsistent correlation coefficients with the Lee’s grading, suggesting that these pathological indexes have differential impacts on the patient prognosis, which should be weighed accordingly when using the Oxford classification system.

IgAN varies greatly in clinical manifestations, pathological changes, and prognosis. Based on the clinicopathological data of 144 patients, we then evaluated the relationship of the MEST-C score with clinical data of patients with IgAN. In the original Oxford classification study, M and S were associated to proteinuria [8]. However, the original Oxford cohort had certain limitations due to the exclusion of some patients with extremely mild or extremely severe IgAN. In this study, all patients regardless of the disease severity were included to understand the relationship between the MEST-C score and clinical parameters.

In a follow-up study of patients with IgAN, Bitencourt-Dia et al. [16] observed that patients with C formation had higher levels of initial proteinuria and Scr than C-free patients. Wang et al. [11] confirmed this finding by observing that proteinuria occurred in all IgAN patients with Cs. In addition, Sasatomi et al. [17] found that IgAN patients with Cs had elevated MAP, and elevated MAP was associated with a poor prognosis of crescentic IgAN. In agreement with these findings, we observed elevated MAP, Scr, and 24 h urine protein but decreased eGFR in patients with crescentic IgAN. Mechanistically, in patients with crescentic IgAN, glomerular epithelial cell proliferation can directly cause podocyte damage and destroy the glomerular filtration barrier, leading to massive proteinuria. Persistent massive proteinuria gradually aggravates renal fibrosis and glomerulosclerosis, thus having a role in the formation of Cs in progressive IgAN [18]. Given that patients with crescentic IgAN usually have proteinuria, elevated MAP, and worsening renal function, it is important to control proteinuria for delaying the disease progression.

In this study, we first used a 50% reduction in ESRD or eGFR as the endpoint to analyze the survival of 144 patients with IgAN. Surprisingly, our results differ from the Oxford cohort study but are in line with most validation studies [19,20], demonstrating that the pathological classifications S, T, and C are prognostic indicators in IgAN. In IgAN, sclerosing and fibrosing processes represent a chronic and irreversible damage that deteriorates renal function and affects the long-term prognosis of patients. For the new C score, numerous studies from different countries have been conducted to verify its performance, but the results are inconclusive. However, at least eight studies in adult patients have proved it to be of prognostic value [12,13,21,22,23,24,25,26], and studies in children with IgAN also found it to be capable of independently predicting the renal outcome [27,28]. Our multivariate Cox analysis results indicate that C is an independent risk factor affecting the prognosis of IgAN, and this conclusion was further confirmed by determining the impact of different C proportions on the prognosis of IgAN.

Our further studies revealed 11% as the cutoff value of C proportions that best reflects the prognosis of patients. Subsequently, we divided all patients into three groups based on the proportion of C formation: 0, <11, and ≥11% and estimated their impacts on the survival and prognosis associated with IgAN by using the Kaplan–Meier method and multivariate Cox regression model. We found that patients with a C proportion ≥11% had a significantly worse survival, supporting the threshold proportion of Cs ≥11% is an independent risk factor for the prognosis of patients with IgAN, which is different from the ≥25% of glomeruli with Cs defined in the 2017 Oxford classification. The reasons for these different results need to be further explored. We speculate that racial differences may be an influencing factor. Our results suggest that Chinese patients with cellular or fibrocellular Cs should receive more aggressive treatments, such as steroids and/or immunosuppressants. It has been reported that mycophenolate mofetil (MMF) treatment is beneficial to the histopathological improvement of IgAN [29]. Additionally, MMF and prednisone or prednisone alone can achieve good treatment response in IgAN patients with active proliferative lesions [30].

However, our study has some limitations. First, we did not specifically evaluate the impact of therapy factors (renin angiotensin inhibitors/angiotensin receptor blockers and immunosuppressive therapy) on the prognosis of patients, which may lead to biased prognostic predictions. Second, IgAN is a chronic progressive disease, but our study only analyzed a short-term outcome of patients diagnosed over a 2 year period. To get more convincing evidence, a validation study with a larger sample size and longer follow-up duration is warranted. Also, multicenter studies involving different populations are needed to assess the predictive values of different proportions of Cs on the prognosis of IgAN.

Considered together, we verified the applicability of the revised Oxford classification and confirmed the clinical significance of cellular or fibrocellular Cs in IgAN patients. Based on these findings, the C score is helpful for the early diagnosis and treatment of IgAN patients. Additionally, the association of the proportion of Cs with the prognosis of IgAN indicate that the C score is a valid classification for predicting renal prognosis. Moreover, we identified a threshold ≥11% is an independent prognostic risk factor for Chinese patients with IgAN. Our results suggest that in clinical practice, even a low proportion of Cs should be paid attention to for early intervention and treatment.

## Abbreviations

ALB: serum albumin
BUN: urea nitrogen
Cs: crescents
E: endocapillary proliferation
eGFR: estimated glomerular filtrating rate
ESRD: end-stage renal disease
HDL: high density lipoprotein
HGB: hemoglobin
IgAN: IgA nephropathy
LDL: low density lipoprotein
M: mesangial hypercellularity
MAP: mean arterial pressure
MMF: mycophenolate mofetil
ROC: the receiver operating characteristic
S: segmental glomerulosclerosis
Scr: serum creatinine
T: tubular atrophy/interstitial fibrosis
TC: total cholesterol
TG: triglycerides
UA: uric acid

## Appendix

**Table A1:** Relationship of M score with clinical parameters of patients with IgAN

Clinical parameter	M0 (*n* = 29)	M1 (*n* = 115)	*P*
Sex (male, %)	9 (31.03)	47 (40.87)	0.332
Age (year)	30.90 ± 12.20	36.31 ± 11.73	0.016*
Hypertension (*n*, %)	4 (13.79)	33 (28.70)	0.101
MAP (mmHg)	93.06 ± 13.16	97.65 ± 13.91	0.102
Scr (μmol/L)	63.61 ± 19.70	103.7 ± 70.5	<0.001*
eGFR (mL/min/1.73 m^2^)	115.5 ± 26.2	84.87 ± 39.78	<0.001*
ALB (g/L)	39.29 ± 7.89	38.53 ± 5.29	0.071
24 h urine protein (g/24h)	0.48 ± 0.49	1.40 ± 1.98	0.009*
BUN (mmol/)	4.54 ± 1.34	5.64 ± 2.82	0.041*
UA (μmol/L)	318.0 ± 102.1	362.2 ± 101.1	0.075
TC (µmol/L)	4.69 ± 2.38	4.50 ± 1.21	0.294
TG (µmol/L)	1.41 ± 0.84	1.88 ± 1.38	0.053
HDL (µmol/L)	1.31 ± 0.35	1.25 ± 0.35	0.372
LDL (µmol/L)	2.69 ± 1.93	2.71 ± 0.84	0.112
HGB (g/L)	130.5 ± 17.1	125.2 ± 17.2	0.142
C3 (g/L)	0.98 ± 0.20	1.02 ± 0.20	0.535
C4 (g/L)	0.24 ± 0.09	0.25 ± 0.08	0.367
IgA (g/L)	2.97 ± 0.87	3.32 ± 1.07	0.123

All quantitative data are expressed as the mean ± standard deviation and categorical variables are expressed as number (percentage). *, statistically significant.

**Table A2:** Relationship of E score with clinical parameters of patients with IgAN

Clinical parameter	E0 (*n* = 102)	E1 (*n* = 42)	*P*
Sex (male, %)	37 (36.27)	19 (45.23)	0.316
Age (year)	33.89 ± 10.39	38.45 ± 14.83	0.194
Hypertension (*n*, %)	20 (19.61)	17 (40.48)	0.009*
MAP (mmHg)	94.80 ± 12.94	101.4 ± 15.0	0.011*
Scr (μmol/L)	91.78 ± 62.55	105.0 ± 72.3	0.091
eGFR (mL/min/1.73 m^2^)	95.23 ± 40.38	80.83 ± 35.04	0.045*
ALB (g/L)	39.65 ± 5.29	36.32 ± 6.62	0.003*
24 h urine protein (g/24h)	0.88 ± 1.36	2.02 ± 2.45	0.002*
BUN (mmol/)	5.16 ± 2.27	6.05 ± 3.28	0.102
UA (μmol/L)	346.3 ± 97.7	370.2 ± 112.7	0.336
TC (µmol/L)	4.38 ± 1.60	4.92 ± 1.19	0.003*
TG (µmol/L)	1.75 ± 1.34	1.87 ± 1.21	0.305
HDL (µmol/L)	1.26 ± 0.35	1.27 ± 0.34	0.862
LDL (µmol/L)	2.66 ± 1.21	2.82 ± 0.94	0.141
HGB (g/L)	126.8 ± 16.2	125.0 ± 19.6	0.563
C3 (g/L)	1.01 ± 0.22	1.01 ± 0.16	0.787
C4 (g/L)	0.24 ± 0.08	0.27 ± 0.09	0.109
IgA (g/L)	3.25 ± 1.06	3.26 ± 1.00	0.848

**Table A3:** Relationship of S score with clinical parameters of patients with IgAN

Clinical parameter	S0 (*n* = 108)	S1 (*n* = 36)	*P*
Sex (male, %)	42 (38.89)	14 (38.89)	0.990
Age (year)	35.61 ± 12.69	33.06 ± 9.62	0.750
Hypertension (n, %)	23 (21.30)	14 (38.89)	0.036*
MAP (mmHg)	95.44 ± 13.52	100.6 ± 14.2	0.065
Scr (μmol/L)	86.89 ± 53.48	121.9 ± 88.7	0.004*
eGFR (mL/min/1.73 m^2^)	97.13 ± 39.37	72.71 ± 33.50	0.001*
ALB (g/L)	38.74 ± 6.34	38.51 ± 4.32	0.188
24 h urine protein (g/24h)	1.05 ± 1.84	1.69 ± 1.67	<0.001*
BUN (mmol/)	5.10 ± 2.33	6.37 ± 3.20	0.010*
UA (μmol/L)	343.3 ± 96.9	383.3 ± 114.0	0.078
TC (µmol/L)	4.42 ± 1.60	4.89 ± 1.13	0.017*
TG (µmol/L)	1.77 ± 1.36	1.81 ± 1.11	0.284
HDL (µmol/L)	1.25 ± 0.35	1.28 ± 0.35	0.613
LDL (µmol/L)	2.64 ± 1.21	2.90 ± 0.89	0.089
HGB (g/L)	125.4 ± 16.6	128.7 ± 19.0	0.332
C3 (g/L)	1.01 ± 0.21	1.01 ± 0.20	0.910
C4 (g/L)	0.25 ± 0.09	0.27 ± 0.07	0.054
IgA (g/L)	3.21 ± 0.97	3.38 ± 1.23	0.676

**Table A4:** Relationship of T score with clinical parameters of patients with IgAN

Clinical parameter	T0 (*n* = 112)	T1/2 (*n* = 32)	*P*
Sex (male, %)	40 (35.71)	16 (50.00)	0.144
Age (year)	34.46 ± 11.08	37.88 ± 11.45	0.102
Hypertension (n, %)	23 (20.54)	14 (43.75)	0.008*
MAP (mmHg)	94.85 ± 13.10	103.3 ± 14.6	0.004*
Scr (μmol/L)	74.09 ± 30.29	171.1 ± 94.8	<0.001*
eGFR (ml/min/1.73 m^2^)	103.4 ± 33.7	47.6 ± 23.6	<0.001*
ALB (g/L)	39.42 ± 5.84	36.10 ± 5.37	0.001*
24 h urine protein (g/24h)	0.81 ± 1.33	2.62 ± 2.50	<0.001*
BUN (mmol/)	4.72 ± 1.50	7.89 ± 3.95	<0.001*
UA (μmol/L)	336.6 ± 89.8	411.7 ± 122.9	0.001*
TC (µmol/L)	4.47 ± 1.56	4.78 ± 1.32	0.079
TG (µmol/L)	1.69 ± 1.18	2.12 ± 1.62	0.041*
HDL (µmol/L)	1.27 ± 0.34	1.24 ± 0.40	0.513
LDL (µmol/L)	2.65 ± 1.19	2.89 ± 0.92	0.074
HGB (g/L)	127.1 ± 16.1	123.1 ± 20.5	0.246
C3 (g/L)	1.02 ± 0.21	0.97 ± 0.19	0.143
C4 (g/L)	0.25 ± 0.08	0.26 ± 0.08	0.223
IgA (g/L)	3.22 ± 0.95	3.35 ± 1.32	0.899
